# Infrared Spectroscopic Electronic Noses: An Innovative Approach for Exhaled Breath Sensing

**DOI:** 10.1021/acssensors.4c02725

**Published:** 2025-01-08

**Authors:** Johannes Glöckler, Jan Mitrovics, Sara Beeken, Marcis Leja, Tesfalem Welearegay, Lars Österlund, Hossam Haick, Gidi Shani, Corrado Di Natale, Raúl Murillo, Gabriela Flores-Rangel, Francisco Bricio-Arzubide, Raul Pinilla, Rómulo Vargas, Carlos Saboya, Boris Mizaikoff, Lorena Díaz de León-Martínez

**Affiliations:** † Institute of Analytical and Bioanalytical Chemistry, Ulm University, Albert-Einstein-Allee 11, 89081 Ulm, Germany; ‡ Breathlabs Inc., Spring, Texas 77386, United States; § Hahn-Schikard, Sedanstrasse 14, 89077 Ulm, Germany; ∥ JLM Innovation GmbH, D-72070 Tübingen, Germany; ⊥ Institute of Clinical and Preventive Medicine, University of Latvia, LV-1586 Riga, Latvia; # Faculty of Medicine, University of Latvia, LV-1586 Riga, Latvia; ∇ Riga East University Hospital, LV-1038 Riga, Latvia; ○ Digestive Diseases Centre GASTRO, LV-1079 Riga, Latvia; ◆ Laboratory for Nanomaterial-Based Devices, Technion – Israel Institute of Technology, Haifa 3200003, Israel; ¶ Department of Electronic Engineering, University of Rome Tor Vergata, 00133 Roma, Italy; ⋈ Interdepartmental Center for Volatilomics, “A. D’Amico”, University of Rome Tor Vergata, 00133 Rome, Italy; ⧓ Centro Javeriano de Oncología, Hospital Universitario San Ignacio, 110231 Bogotá, Colombia; ⧖ Facultad de Medicina, Pontificia Universidad Javeriana, 110231 Bogotá, Colombia; ● Department of Materials Science and Engineering, The Angstrom Laboratory, Uppsala University, 752 37 Uppsala, Sweden

**Keywords:** gastric cancer, IR spectroscopy, iHWG, IR sensor, mid-infrared, MIR, electronic nose, eNose, MOX sensors, exhaled breath, exhalome, volatile organic compounds, VOCs

## Abstract

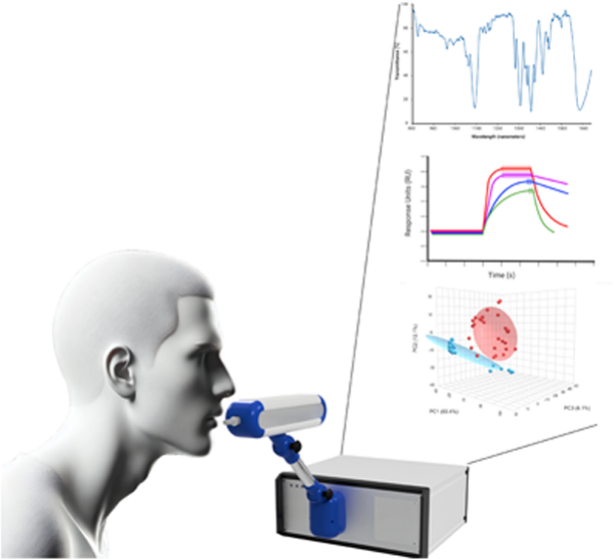

Gastric cancer remains a leading cause of cancer-related mortality, requiring the urgent development of innovative diagnostic tools for early detection. This study presents an integrated infrared spectroscopic electronic nose system, a novel device that combines infrared (IR) spectroscopy and electronic nose (eNose) concepts for analyzing volatile organic compounds (VOCs) in exhaled breath. This system was calibrated using relevant gas mixtures and then tested during a feasibility study involving 26 gastric cancer patients and 32 healthy controls using chemometric analyses to distinguish between exhaled breath profiles. The obtained results demonstrated that the integration of IR spectroscopy and eNose technologies significantly enhanced the accuracy of VOCs fingerprinting via principal component analysis (PCA) and partial least-squares-discriminant analysis (PLS-DA). Distinct differences between the study groups were revealed with an accuracy of prediction of 0.96 in exhaled breath samples. This combined system offers a high sensitivity and specificity and could potetially facilitate rapid on-site testing rendering the technology an accessible option for early screening particularly in underserved populations.

Gastric cancer remains a public health concern due to its significant global burden, as the fourth leading cause of cancer-related deaths worldwide. In 2021 alone, 954,373.6 (821,750.81–1,089,576.58) deaths associated with this condition were reported.^1^ Gastric cancer develops in the lining of the stomach, typically beginning in the cells that produce mucus in the innermost layer of the stomach as well as in other gastric fluid-producing cells. The most common form of this cancer is adenocarcinoma, accounting for 90–95% of cases.^2^ In its early stages, gastric cancer may present with mild symptoms or even be asymptomatic; and, symptoms such as nausea, vomiting, frequent stomach pain, loss of appetite, intentional weight loss, bloating, difficulty swallowing, and blood in the stool are usually associated with advance disease. The causes of gastric cancer, as with other cancers, are multifactorial and include risk factors such as chronic *Helicobacter pylori* infection, frequent tobacco and alcohol consumption, diets high in sodium and processed foods, gastric ulcers, chronic gastritis, and, in some cases, genetic predisposition.^3^ In this context, early detection is vital to achieving better patient outcomes as well as improved quality of life for those affected. Currently, the diagnosis of gastric cancer involves various approaches, including endoscopy (esophagogastroduodenoscopy, EGD), biopsy, endoscopic ultrasound, barium swallow (upper gastrointestinal series), computed tomography, magnetic resonance imaging, positron emission tomography, and laparoscopy.^4^ Most of these procedures are invasive, have high associated costs, and require highly trained personnel, making them difficult to use in mass screening settings, especially in underserved populations with limited access to healthcare services, as is the case in many low- and middle-income countries. In this context, Colombia is a middle-income Latin American country, and gastric cancer is currently the leading cause of cancer-related deaths in the country, with about 7000 deaths per year.^5^ The high mortality is primarily associated with the late detection of the disease, resulting in a significant economic burden, not only for affected families but also for the healthcare system as a whole. In Colombia, the financial burden of gastric cancer has increased substantially in recent years. Cost-effectiveness varies depending on the stage at which the disease is diagnosed; for instance, in early stages (0-IA), the approximate treatment cost is 22,434 EUR, whereas in more advanced stages (IIIA–IIIB), treatment costs range between 22,445 and 23,498 EUR, but survival is substantially different between early and advanced stages.^6^ Despite the severity of the disease, efforts for early detection, prevention, and awareness are still insufficient. In Colombia so far, mass screening programs are nonexistent, which contributes to this increasing rate of late detection.

In this context, research focused on the development and application of sensitive, specific, and accessible methodologies for the screening and monitoring of gastric cancer progression is of critical importance. In recent decades, the study of exhalome (exhaled breath analysis) has gained significant relevance in biomedical research as “we exhale who we are and how we are.” The composition of exhaled breath can provide valuable information regarding an individual’s metabolic state and health status, as it reflects their physiological and pathophysiological status at a given time. This makes it a subject of interest in the medical and clinical fields.^7,8^ Exhaled breath is a complex mixture of gases released during respiration, primarily composed of CO_2_ (∼3.6%), water vapor (∼6.5%), O_2_ (∼15.7%), N_2_ (∼74.9%), trace amounts of other gases such as Ar and He (∼0.6%), and volatile organic compounds (VOCs).^9^ These compounds originate from various metabolic processes and have high solubility in blood and high volatility and are capable of being transported through the bloodstream and filtering into saliva, making them detectable in exhaled breath. To date, over 3000 VOCs have been identified in exhaled breath, many of which have been linked to the development of various diseases, importantly in gastric cancer. They provide a noninvasive way to monitor health conditions and could serve as biomarkers for disease diagnosis and progression.

Various studies have linked specific VOCs to the disease, highlighting their potential as noninvasive diagnostic tools. These include hexanoic acid, phenol, methyl phenol,^10^ 2-propenenitrile, furfural, 6-methyl-5-hepten-2-one, styrene, 2-ethyl-1-hexanol, nonanal,^11^ hexadecane, butyric acid, pentatonic acid, decanal,^12^ acetone, isoprene, 1,3-dioxolan-2-one, phenol, meta-xylene, 1,2,3-trimethylbenzene, phenyl acetate,^13^ ethylene, methyl isobutyl ketone, acetic acid, *m*-toluyl aldehyde.^14^ VOCs are usually assessed through analytical techniques such as GC-MS, SIFT-MS, PTR-MS, and IMS. Nevertheless, these techniques present challenges related to sample collection and processing, prolonged analysis times, and the need for highly trained personnel for its execution, resulting in high associated costs. In this scenario, we propose the first combined infrared (IR)-spectroscopic electronic nose (IR-eNose) system for label-free noninvasive exhaled breath analysis.

eNoses are highly sensitive devices that mimic the mammalian sense of smell (olfactory system) to detect and identify complex mixtures of VOCs in gas-phase samples. They usually consist of sensor arrays designed to respond to different groups of molecules and generate a signal related to the presence and concentration of these compounds.^15^ Some studies have shown among other things^16−19^ that a stand-alone measurement principle (i.e., sensor array) is not sufficient to fully capture the complex composition of the exhalome, especially with regard to diagnostically relevant, disease-specific biomarkers combinations, and even with their high sensitivity, eNoses, present disadvantages due to their limited specificity, which hinders the accurate distinction between chemically similar compounds, leading to cross-sensitivity and reducing accuracy in complex matrices, such as exhaled breath. Additionally, the available VOC databases for pattern comparison are limited, which affects their performance. Considering this, gas-phase IR spectroscopy has substantial relevance. VOCs are detected and quantified via the absorption of IR radiation at specific wavelengths especially within the so-called “fingerprint” region of the mid-infrared spectrum (3–15 μm). Molecules in the exhaled breath matrix absorb IR radiation at characteristic frequencies corresponding to their vibrational, rotational–vibrational and rotational transitions.^16^ This absorption creates a unique spectral fingerprint, allowing the identification and quantification of specific compounds. Gas-phase IR spectroscopy presents advantages such as high specificity for the identification of functional groups of molecules, providing both quantitative and qualitative data on target compounds.

In this study, we demonstrate a portable IR-eNose system optimized for exhaled breath analysis that combines both orthogonal analytical approaches providing high sensitivity via the eNose and high specificity via IR for the advanced detection and assessment of complementary patterns associated with the same exhaled breath sample. This could potentially allow early gastric cancer condition assessment beyond clinical settings.

## Materials and Methods

An IR-eNose system was designed, developed, and tested specifically for exhaled breath analysis. After calibration and laboratory testing, this system was applied in a real-world clinical scenario at three different medical centers in Colombia by screening volunteer gastric cancer patients and healthy controls.

### IR-eNose System Description

**Figure 1:**
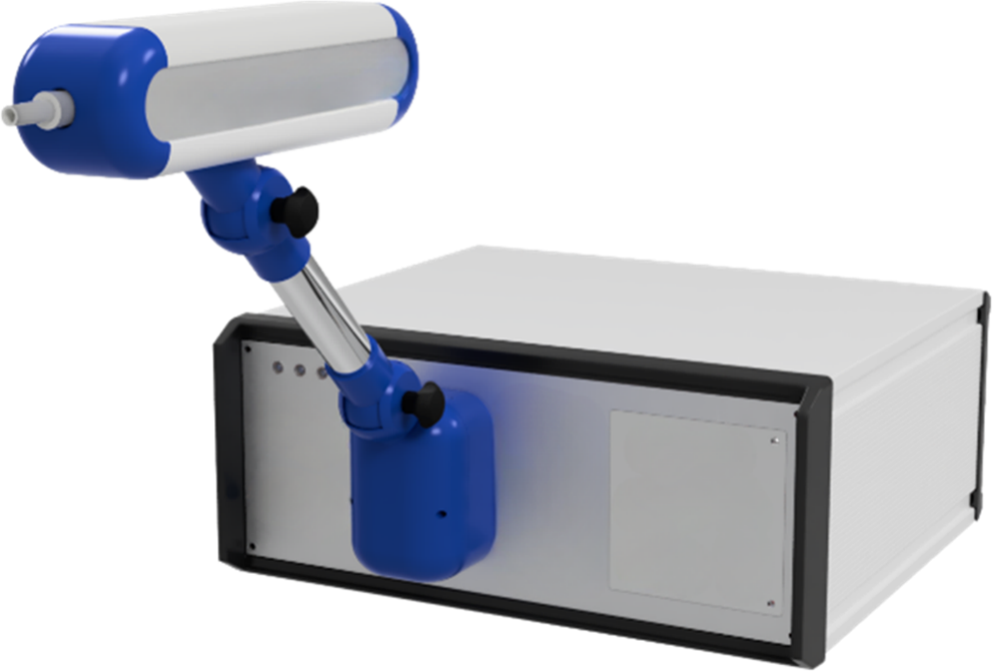
Combined IR-eNose system with breath collection module providing direct access to the patient.

**Figure 2:**
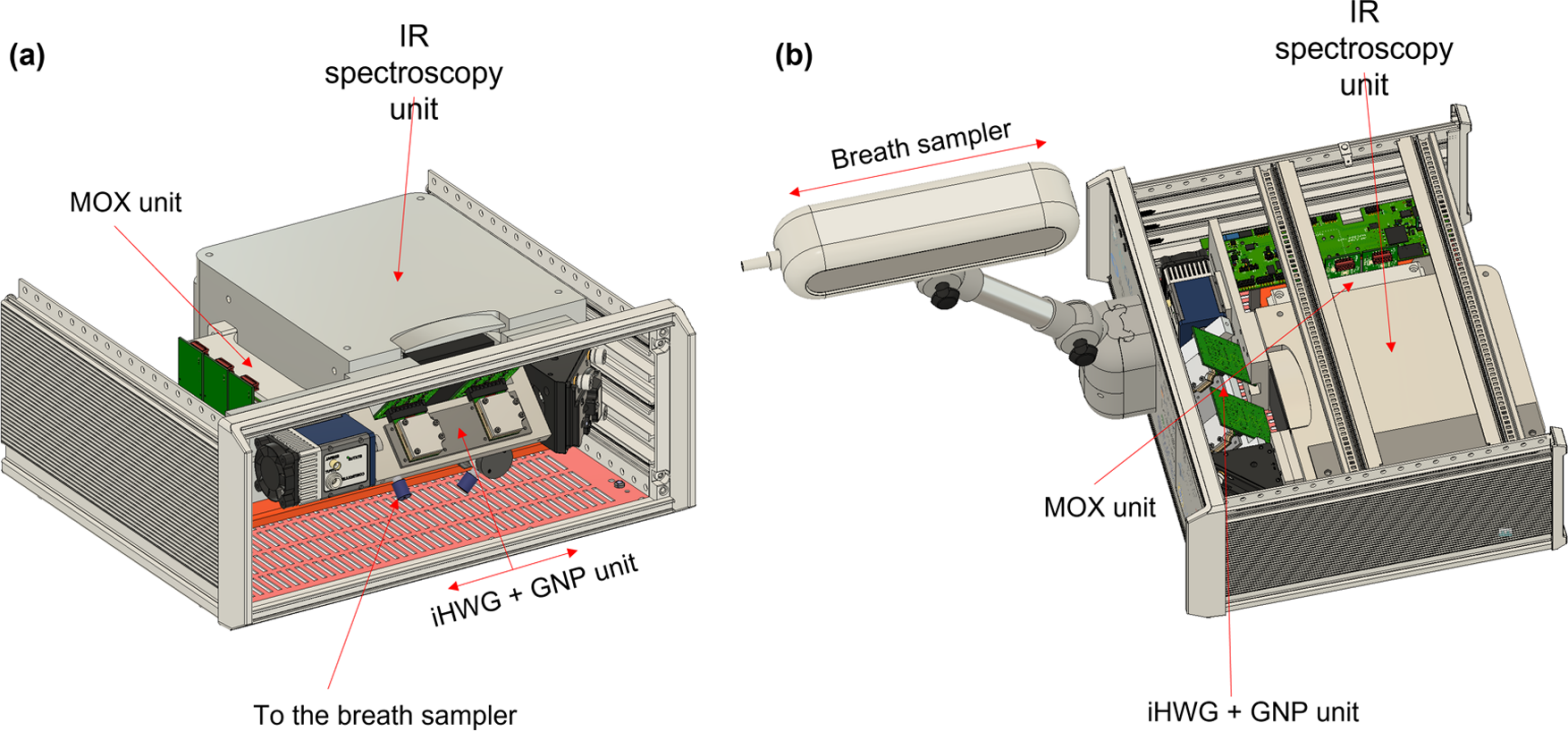
Rendering of the open device: (a) infrared (IR) spectroscopy unit, metaloxide (MOX) unit, GNP (gold nano particle) unit, substrate-integrated hollow waveguide (iHWG), and (b) breath sampler unit.

The IR-eNose system was composed of three main modules. The breath sampler, the eNose, and the IR sensing system (Figures 1 and 2).

### Breath Sampler Module

The breath sampler module was based on the buffered-end-tidal sampling process by which the end-tidal portion of exhalation was collected and then sent to the sensing chamber. It consisted of an exchangeable mouthpiece, a temperature-stabilized PEEK glass tube to keep the exhaled breath at body temperature to avoid condensation (37–40 °C), a controlled system of pump and valves to drive the part of the exhaled breath of interest to the sensing system, and a fan to clean the breath tube. It was mounted on a short arm and connected to the main unit through cables and a heated tube running inside the arm (Figure 2a).

### eNose Module

The eNose module comprised two main sensor units, one equipped with metal oxide sensors (MOX) and another one with gold nanoparticle (GNP) sensors. The MOX unit comprised several modules: (i) one module with 8 commercial analog MOX sensors, (ii) a module with 8 commercial digital MOX finally providing a total of 14 individual sensor signals (Table S1), and (iii) three experimental MOX sensors which are based on nanoporous polycrystalline NiO films deposited on alumina substrates developed by Uppsala University, Sweden; these sensors were introduced in order to test their performance in exhaled breath analysis.^20,21^ As for the GNP sensors unit, each one was equipped with 24 sensors (6 replicas and 4 chemistries). These sensors were developed by Technion Institute, Israel and were enhanced by using eight organic ligands of different functional chemistry, which are described in several publications.^22−24^ This module was based on a previous developed system reported elsewhere^25^ (Figure 2b). Hence, in the present study, only the MOX sensor readings were evaluated.

### IR Spectroscopy Module

The IR spectroscopy module was developed around the key component of the entire systema specially designed substrate-integrated hollow waveguide (iHWG)—, a technology pioneered by Ulm University, Germany.^16,26−29^ The system (Figure 2b) comprised an (i) FTIR spectrometer module (Bruker α II, Bruker Optics GmbH, Ettlingen, Germany) with a thermoelectrically cooled mercury–cadmium–telluride (MCT) detector (MIP-10-1M-F-M4, VIGO Systems S.A., Poznanska, Poland) operated at a spectral resolution of 1 cm^–1^ and (ii) an iHWG with a straight waveguiding channel (15 cm long) serving both as a miniaturized gas cell and as a light-transmitting photon conduit made from 6082-grade aluminum.

The custom-designed iHWG consisted of a cylindrical channel with a diameter of 4 mm yielding a probed gas sample volume of 1.9 mL. The iHWG channel was sealed by IR-transparent zinc selenide windows at each end, facilitating in- and out coupling of infrared radiation (Figure 2b). On the in-coupling side of the waveguide, a mirror mount (Thorlabs KCB1P/M, Thorlabs GmbH, Dachau/Munich, Germany) was located, which held a 1″ (37-241, Edmund Optics GmbH, Karlsruhe, Germany) off axis parabolic mirror (OAPM). Due to the specific design of the iHWG, the infrared radiation emanating from the FTIR spectrometer is reflected by the OAPM and focused onto the entrance facet of the iHWG. At the distal end of the iHWG, the detector was directly attached to the output facet of the iHWG; thus, no additional optics were needed, and an alignment-free, robust, and highly efficient optical assembly was ensured.

### System Integration

**Figure 3:**
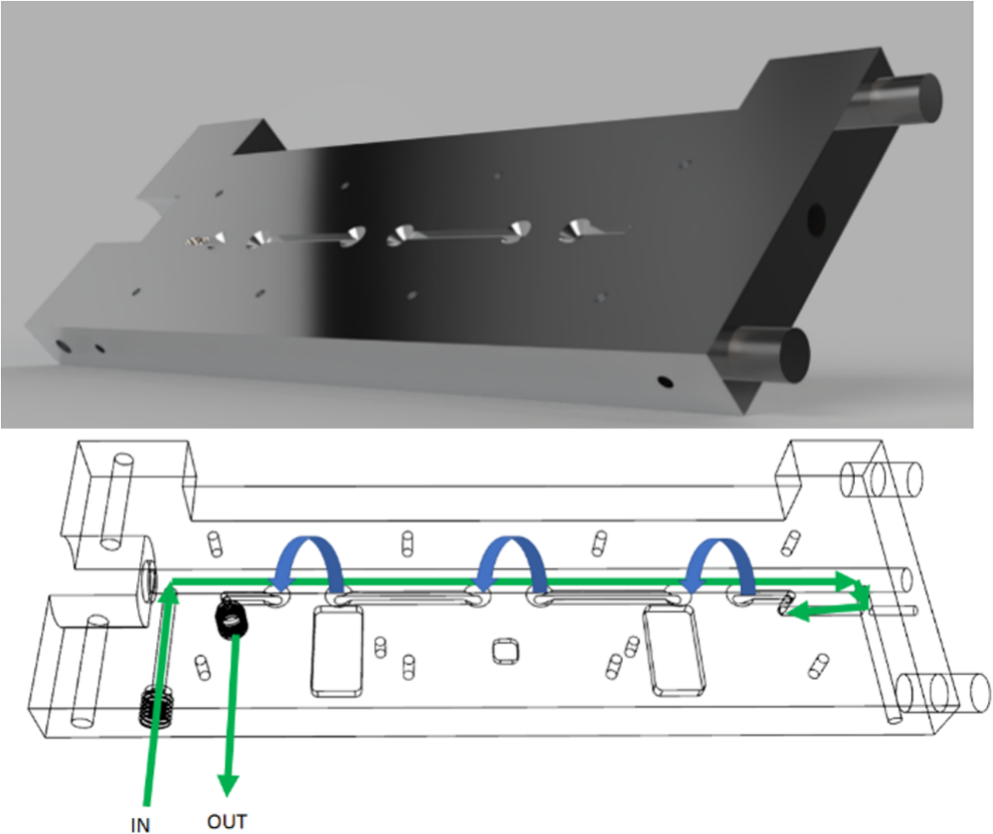
iHGW with integrated MOX/GNP sensors. The pathway of the gas/sample during measurement is illustrated. The breath sample is introduced into the iHWG-eNose structure and first passes through the iHWG for recording an IR spectrum. At the end of the iHWG channel, the gas flow is guided to the MOX/GNP gas chambers. The MOX chambers are not shown for the sake of clarity.

In order to reduce the dead volumes and optimize the connection pathways between the IR module and the eNose, the sensor modules were integrated into the iHWG structure. Briefly, the breath sample is introduced into the iHWG-eNose structure and first passes through the iHWG chamber, where IR radiation interacts with the molecules to yield an IR absorption spectrum. At the end of the iHWG channel, the gas flow is guided to the MOX/GNP gas chambers. Hence, from the same breath sample, the IR spectra and the sensor signals are simultaneously recorded and inherently synchronized (Figure 3).

### IR-eNose System Assessment

#### Operation Parameters

The sensor boards were consistently maintained at a temperature of 40 °C throughout the entire analysis process. Initially, a measurement of ambient air was taken to establish the baseline for the sensors and the spectrometer. This background measurement ensured that any subsequent changes in the detected compounds would be attributed to the exhaled breath and not environmental variables.

After the baseline was set, patients were instructed to exhale through the mouthpiece of the exhaled breath sampler, enabling measurement of their exhaled breath. Following a cleaning step was performed to make sure the system was completely back to baseline, to purge any remaining compounds from the system, preventing cross-contamination between patient samples. In addition a disposable mouthpiece was used for each patient.

IR spectra were collected across a spectral range of 4000–700 cm^–1^ with a resolution of 2 cm^–1^. A Black-Harris 3-term apodization function was applied to improve the signal, and 64 scans were averaged for each measurement to enhance the data quality. The IR data acquisition was managed using OPUS 8.5 software (Bruker Optik GmbH, Ettlingen, Germany), with a custom script developed to synchronize the sensor data and IR data into a single file. The device was equipped with an internal computer, and the data were saved in a .JSON file format for further analysis.

#### Calibration Functions

In order to assess the proper functioning of the IR-eNose system, a series of calibration functions were recorded using gas standards of known concentration including acetone (ACE), acetaldehyde (ACH), *n*-pentane (N-PEN), and nitric oxide (NO) simulating the presence of various biomarkers in exhaled breath.

For the gas supply, a system consisting of Bronkhorst mass flow controllers (MFCs) with varying flow capacities were utilized for each gas. The MFCs were automatically controlled using a custom-developed LabVIEW program to precisely regulate the calculated flow rates, yielding accurate gas mixtures. The total gas flow rate was maintained at a rate of 500 mL/min throughout the analysis. The sample collection parameters were the same as those previously described.

#### Breath Sampling: a Gastric Cancer Screening Feasibility Study

A group of 26 volunteer gastric cancer patients and 32 volunteer controls were recruited at the Colombian National Cancer Institute, Centro Javeriano de Oncología-Hospital Universitario San Ignacio and Community Endoscopy Centre María Auxiliadora for an experimental study within the European Union project VOGAS (project # 824986; https://www.vogas.eu/about/). The sampling was conducted under ethical standard compliances, and the study was registered in the ClinicalTrials.gov Protocol Registration and Results System (protocol ID: 824986, ClinicalTrials.gov ID: NCT04022109) and approved by the Ethics Committee at the Hospital Universitario San Ignacio (FM-CIE-0056-19).

For sample collection, participants were instructed to fast overnight and abstain from smoking, consuming alcohol, chewing gum, or engaging in physical activity for at least two hours before providing the breath sample and to avoid the use of perfumes and fabric softener until the sample collection. Eligibility criteria included for the gastric cancer group: (i) patients with histopathological diagnosis of gastric cancer; (ii) without prior surgery and chemotherapy; (iii) without any other oncologic malignancies; (iv) without recent infections under antibiotic treatment; and (v) without serious comorbidities. For the control group, (i) subjects without gastric cancer or any other oncology malignancies; (ii) without evidence of precancerous lesions; (iii) without recent infections under antibiotic treatment; and (iv) without serious comorbidities. All patients were asked to sign informed consent.

Prior to sampling, each participant received detailed instructions and a demonstration of the breath collection protocol. No lung-wash-out was carried out prior or during the sampling; the patients were instructed to perform a mouth rinse with distilled water to reduce the external pollutants, standardize sampling conditions, minimize bacterial influence and saliva components and hydration of the oral cavity, and hydrate the mucous membranes, which may reduce the concentration of VOCs originating from dry or dehydrated oral tissues; all of these parameters to provide a more stable baseline for analysis. During the sampling procedure, participants were seated and asked to take some deep breaths, in order to normalize the lungs’ VOC composition, to then blow into the device. Breath samples were collected by having participants exhale directly into the device’s inlet for a minimum of 5 s. The device featured integrated sensor technology that provided feedback to ensure that the sample was adequate. If the breath collection was insufficient, participants were prompted to repeat the procedure. Baseline measurements were taken for standardization across all samples.

### Data Evaluation

#### IR Data Preprocessing

A background extraction was performed in order to reduce the contribution of moisture to the IR spectra. After a baseline correction, a 21-point Savitzky-Golay smoothing was applied to all spectra to remove noise. The CO_2_ bands were also corrected. Spectra processing was achieved by Essential FTIR, ORIGIN Pro, and Orange Quasar. For each individual calibration gas, also in the exhaled breath matrix, selected wavelength ranges provided thefingerprint information, as summarized in Table 1.

**Table 1:** Selected IR Wavelength Ranges for Individual VOCs of Interest

Gas sample	Abbreviation	Wavelength ranges (cm^–1^)
acetone	ACE	1700–1100
acetaldehyde	ACH	1700–1100
*n*-pentane	NPEN	3200–2400
nitric oxide	NO	2100–1400
exhaled breath	EB	2400–1200

#### eNose Data Preprocessing

Data from all sensors were extracted, only the 28 most reproducible MOX sensors were analyzed for this purpose. The reproducibility of the sensors was analyzed through their response to the calibration gases; only the ones with the coefficient of variability between 5 and 10% were considered for the subsequent analysis. All data were normalized using a fractional difference model: Δ*R*/*R*_0_ = (*R*_max_ – *R*_0_)/*R*_0_, where *R* is the response of the system to the sample gas, and R_0_ is the baseline reading from ambient air. An autoscaling was carried out to eliminate the effects of the magnitude of the sensor responses.

#### Chemometric Data Analysis

A chemometric approach was developed for evaluating the patterns related to the exhaled breath samples first for each technology individually and then for fused data sets. A principal component analysis (PCA) was applied as an unsupervised method; this technique is used to reduce the dimensionality of the data set while preserving as much variance as possible. PCA derives a set of uncorrelated variables known as principal components from the original variables. These principal components represent linear combinations of the initial variables, whereby the first principal component captures the largest variance within the data set. Subsequent components account for progressively lower variance while maintaining orthogonality with the preceding components. PCA achieves dimensionality reduction by identifying a subset of these components that capture the majority of variability within the data, thus simplifying and facilitating the analysis of intricate data sets.^30^ Afterward, partial least-squares-discriminant analysis (PLS-DA) was used as a supervised methodology in order to achieve classification by modeling the relationship between predictor variables and categorical response variables. PLS-DA combines the dimensionality reduction capabilities of PLS regression with the discriminative power needed for classification models. PLS-DA projects both the predictor and response variables into a new latent space, maximizing the covariance between them while simultaneously distinguishing between predefined classes in response variables. Thus, the discrimination between groups in a data set is optimized.^31^ PLS-DA was then validated via cross-validation to obtain the accuracy of classification of each model. Then, random forest classifier (RFC) was applied. This method is based on the aggregation of multiple decision trees (500 in this case) operating by constructing several decision trees into a training set, where each tree is built by a random subset of features and samples from the data set. This reduces overfitting and increases the model robustness. RFC is particularly effective in handling large data sets with high-dimensional features, noisy data, and missing values, while providing important insight via feature importance scores.^32^ All data analyses were performed using Orange Quasar software and MetaboAnalyst 6.0 statistical software (https://metaboanalyst.ca).

The cross-validation methodology employed in this investigation utilized a *k*-fold partitioning strategy, wherein the model was iteratively recalibrated for each fold to ensure the strict segregation of training and validation cohorts. This process involved the systematic division of the data set into mutually exclusive subsets, with one subset designated as the validation set per iteration, while the amalgamation of remaining subsets constituted the training set. This rigorous approach precludes any contamination of the validation data in the model construction phase, thereby yielding an unbiased estimation of the model’s predictive efficacy. The implementation of this cross-validation protocol not only corroborates the model’s robustness but also substantiates its generalizability, underscoring its discriminative capacity in delineating the study cohorts. Furthermore, this methodological framework mitigates the risk of overfitting and provides a more reliable assessment of the model’s performance on unseen data, thus enhancing the validity and reproducibility of the findings.

## Results and Discussion

### Integrated IR-eNose System

The IR-eNose system was successfully optimized, and a total of six prototype systems were built, which are currently used in clinical studies at several locations worldwide. This ground-breaking and unique combination of orthogonal technologies applied to studying the human exhalome is reported herein for the first time as a fully integrated, portable, and field-deployable system. The IR-eNose system is tailored and optimized for effectively, sensitively, and selectively analyzing VOCs patterns present in exhaled breath with potential applications beyond clinical settings as an assistive tool for screening and monitoring of gastric cancer in addition of other diseases characterized by assignable VOCs exhaled breath patterns (Figure S1). It is important to note that first, a validation of the separate technologies (i.e., eNose and IR-iHWG spectroscopy) was performed in order to assess the proper device function prior to the integration. The results of the calibration gases are presented for the individual technologies to avoid bias during the proof-of-concept phase and prior to the in-field feasibility study analyzing the exhaled breath of gastric cancer patients and healthy controls.

### IR Data

**Figure 4:**
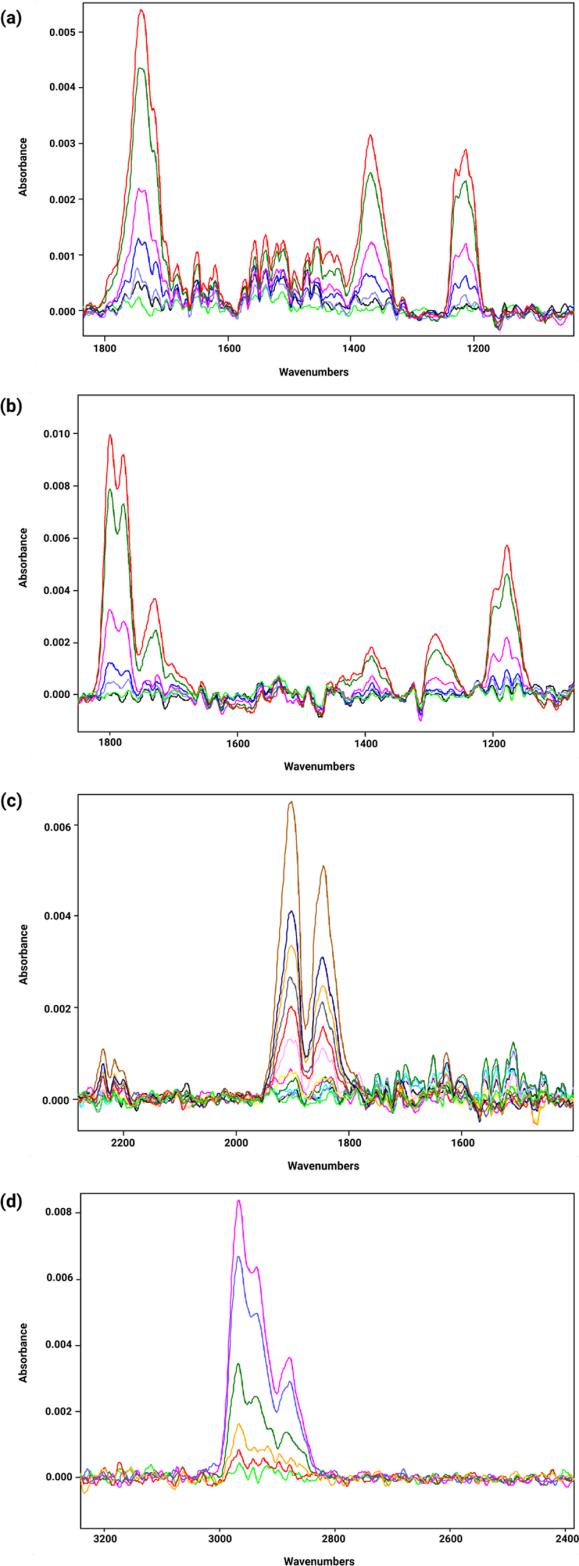
IR spectra obtained for (a) ACE, (b) ACH, (c) NO, and (d) N-PEN and the related calibration functions.

**Figure 5:**
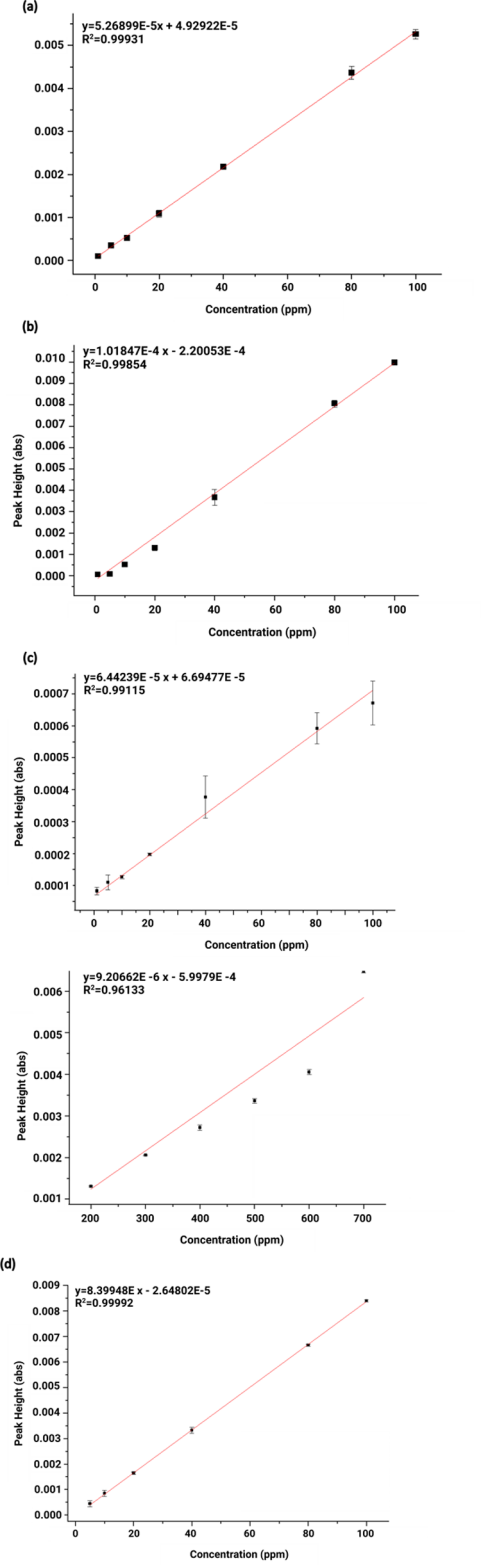
Calibration functions derived from the IR spectra of each analyzed gas: (a) ACE, (b) ACH, (c) NO, and (d) N-PEN.

The following section presents IR spectra and calibration functions generated for each of the selected gases. For ACE, the obtained IR spectrum exhibited a characteristic peak in the range 1800–1600 cm^–1^, which was selected for further evaluation. Calibration functions were generated considering an ACE concentration range of 1–100 ppm (Figure 4a). The results and the calibration function for ACE are shown in Figure 5a including the parameters and their respective linear range. For ACH, the IR spectrum displayed a characteristic peak in the range of 1240–1137 cm^–1^, which was chosen for subsequent evaluation in the calibration functions, which were generated over a concentration range of 1–100 ppm (Figure 4b). The results and the corresponding calibration function are presented in Figure 5b including the equation parameters and the associated linear range. For NO, a linear range of 1–700 ppm was considered due to the compound’s low absorbance, and for this reason, two separate calibration functions were created: one for 1–100 ppm and another for 200–700 ppm to maintain linearity and sensitivity across different concentration ranges (Figure 5c). The characteristic peak used for the calibration functions was in the range 1950–1890 cm^–1^ (Figure 4c). Finally, for *n*-pentane (N-PEN), the selected wavelength range for calibration was the characteristic peak observed in the range of 3000–2950 cm^–1^ (Figure 4d) with a linear range of 5–100 ppm. The corresponding calibration function and associated parameters are listed in Figure 5d. It is important to note that for this evaluation, the peak heights were analyzed in units of absorbance, as described above.

### eNose Data

This section presents the results related to the detection of the calibration gases using eNose with the objective of evaluating the functionality of coupling the sensor chamber to the waveguide and utilizing the same sample for both detection technologies.

**Figure 6:**
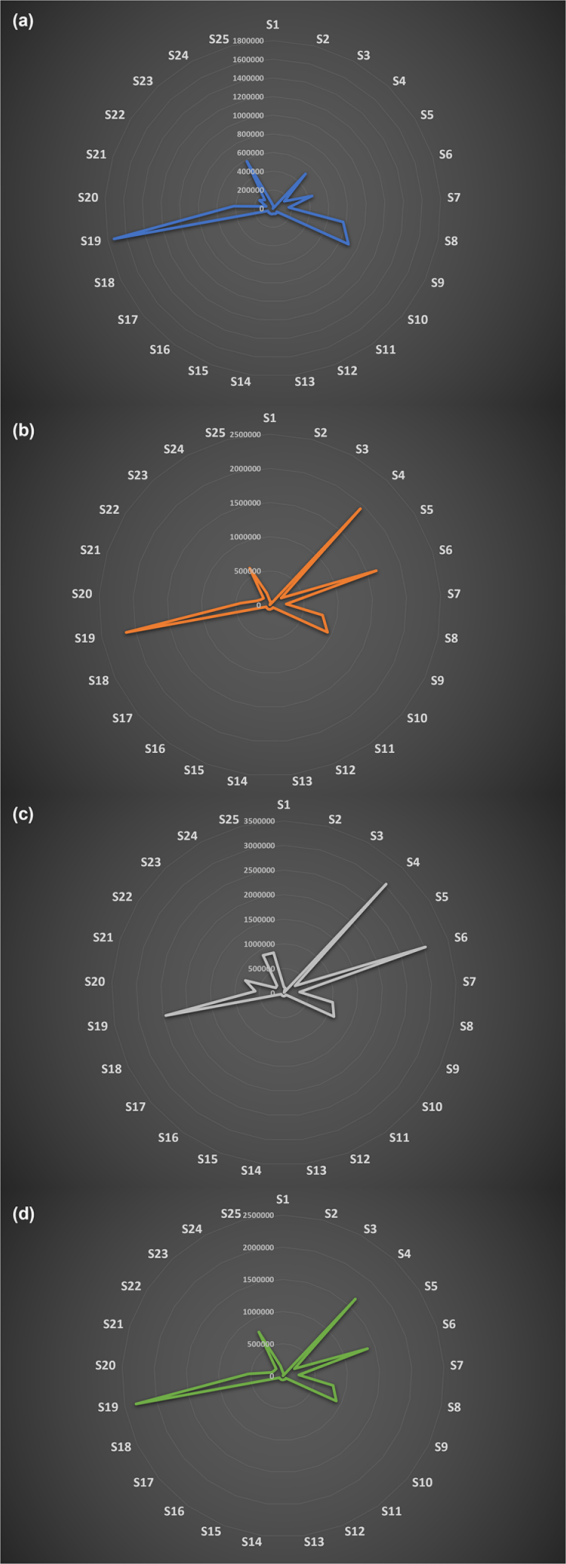
eNose sensor patterns for each calibration gas. (a) ACE, (b) ACH, (c) NO, and (d) N-PEN.

Only the responses from the MOX sensors were evaluated in the present study, as they demonstrated the highest stability and linearity with respect to the calibration gas responses that were considered (i.e., a total of 25 responses, each treated as a separate sensor). Figure 6 shows the 25 responses of the sensors included in the present analysis for ACE, ACH, NO, and N-PEN. The distinct response patterns (i.e., “eNose fingerprints”) of the sensors to the different gases are evident, demonstrating the efficiency of the technology for the selective detection of each calibration gas. It is important to note that the eNose is indeed highly sensitive particularly at low concentrations resulting in linear responses for all compounds within the 1–20 ppm range. Beyond these concentrations, saturation is observed for the sensors. The results for the sensor calibration are summarized in Tables S2–S5. The intercept, slope, correlation, and the determination coefficients are presented for each sensor. It is immediately evident that all sensors revealed a linear response with an average *R*^2^ > 0.80.

### Chemometric Data Analysis

#### Calibration Gases by eNose

**Figure 7:**
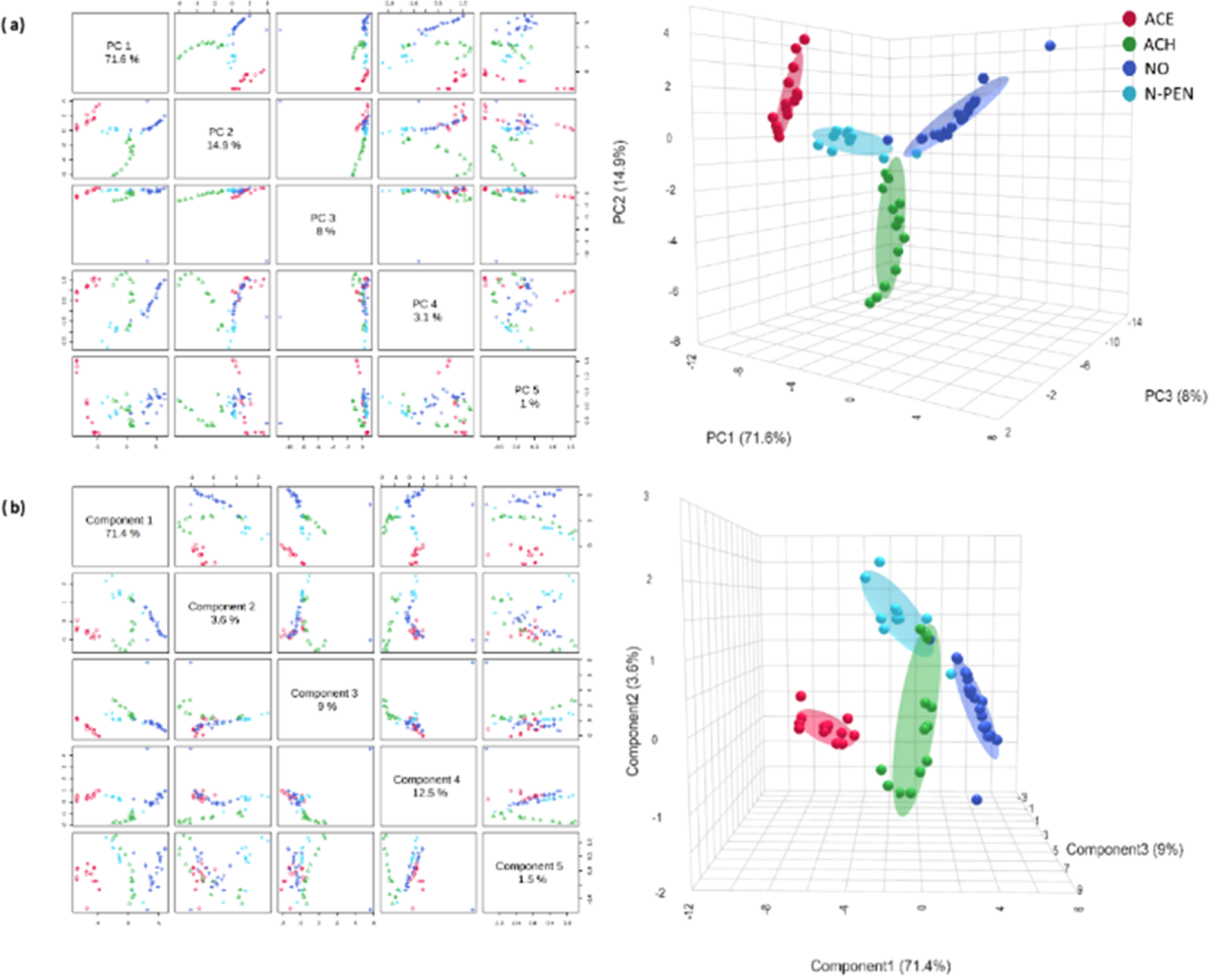
(a) Principal component analysis (PCA) and (b) partial least-squares-discriminant analysis (PLS-DA) from the calibration model.

This section presents the chemometric analysis related to the validation tests of the eNose. Chemometric models were constructed by using the responses from each sensor. The results from PCA demonstrate the differences between the fingerprints of the various calibration gases as well as a clear discrimination between groups. The PCA explained an accumulated variability of 98.1% across five principal components (PC1 = 71.6%, PC2 = 14.9%, PC3 = 8%, PC4 = 3.1%, and PC5 = 1%), with 94.5% of the variance explained by the first three components (Figure 7a). PLS-DA maximized the differences between the groups of each calibration gas (Figure 7b), accounting for 98% of the variability among groups through five components. The cross-validation results of the PLS-DA indicate a high model performance, with an accuracy of 0.92 (where 1 is the maximum value) using five components (Table S6). The RFC was applied to validate the model as predictive; 500 trees were selected. The out-of-bag (OOB) error, which is an estimate of the error rate (which is 1 – accuracy) that this training approach has for new data from the same distribution, was 0.0469, which demonstrated the correct predictive ability from the calibration model as well as the high associated accuracy (Table S7), with the higher error presented between the N-PEN and the NO classification.

#### Exhaled Breath Feasibility Studies for Gastric Cancer Screening via the IR-eNose System

**Figure 8:**
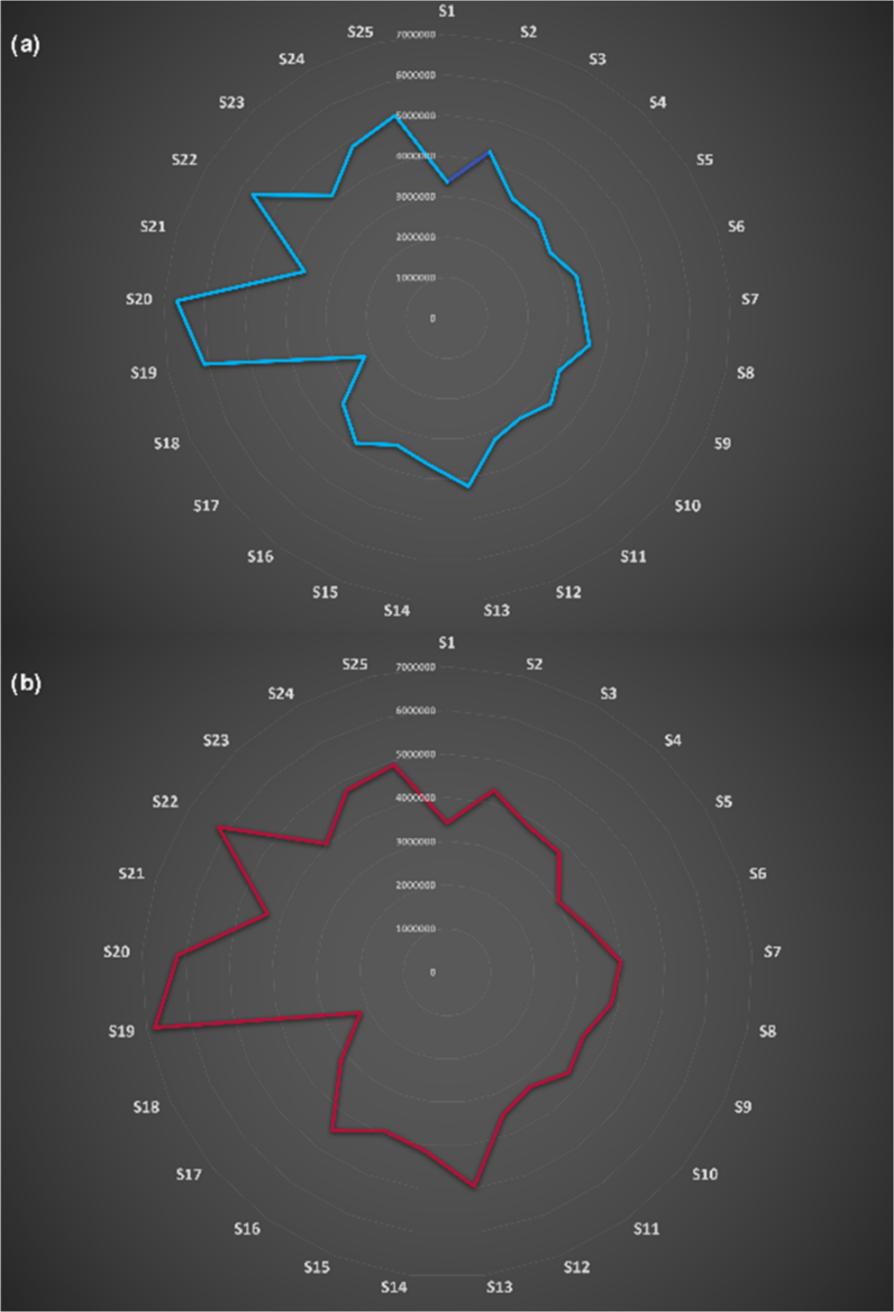
Patterns of chemical fingerprints derived from the eNose sensing associated with exhaled breath samples. (a) Patients with gastric cancer and (b) healthy participants.

In this section, chemometric analysis evaluating the utility of the IR-eNose system for analyzing the exhaled breath from patients with gastric cancer is presented. For this purpose, a total of 58 participants were recruited, including 26 gastric cancer cases and 32 healthy subjects, who met the inclusion criteria. Regarding the differences in the exhaled breath patterns between both study groups obtained via eNose sensing, clear differences were observed, which are illustrated in Figure 8.

Regarding the IR spectra, the differences between the spectra of the study groups are not immediately apparent and humidity significantly affects the spectral region between 2400 and 1200 cm^–1^ despite efforts to remove the rotational lines associated with water. Therefore, multivariate statistical analysis was conducted to uncover potential characteristic patterns for each study group and identify possible differences. Additionally, the most relevant features for group discrimination were selected. To achieve this, a *t* test was applied to determine whether the spectral differences between groups were statistically significant. A total of 826 features were found to be statistically significant (*P*-value < 0.01), and the results are presented in Figure S2.

**Figure 9:**
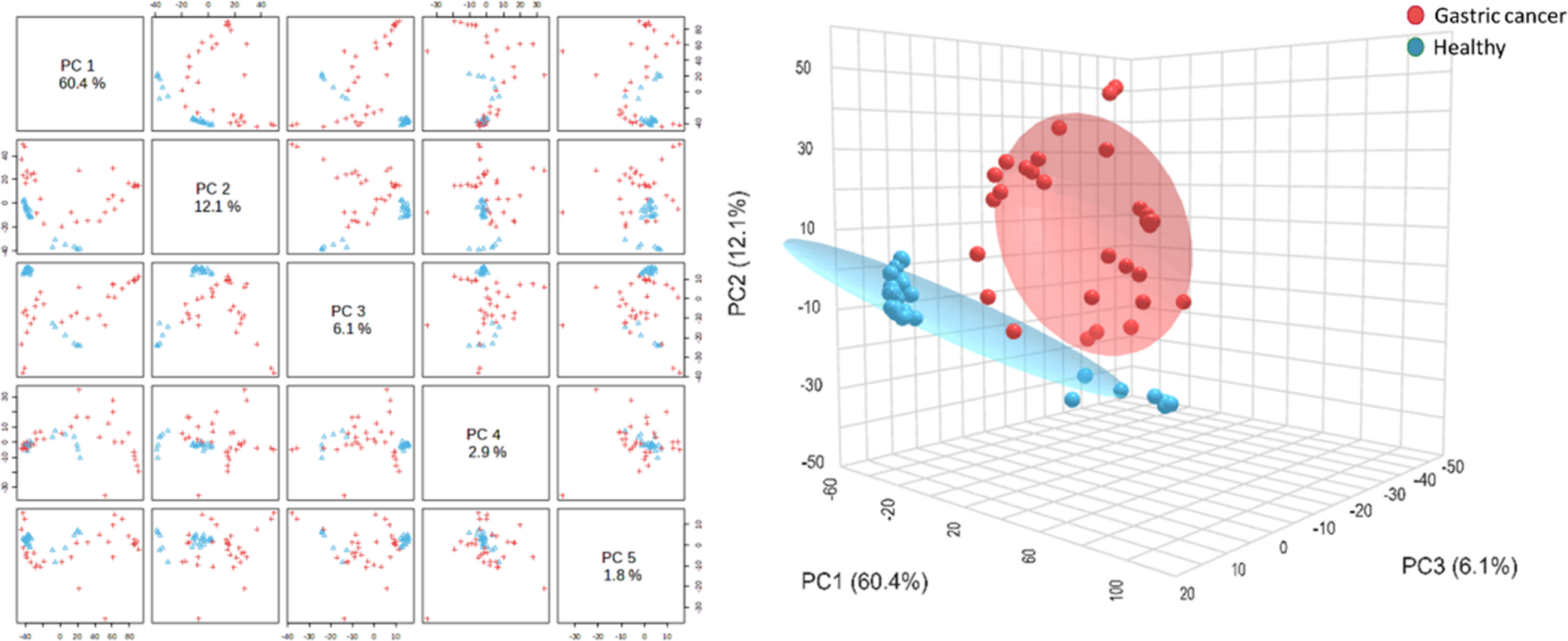
Principal component analysis (PCA) for the evaluation of the IR-eNose system performance in exhaled breath from gastric cancer patients and healthy participants.

Concerning the chemometric analysis, the PCA results using fused data from both technologies demonstrate clear differences between the study groups with an accumulated variance of 83.3% explained by five PCs (PC1 = 60.4%, PC2 = 12.1%, PC3 = 6.1%, PC4 = 2.9%, and PC5 = 1.8%), indicating a high discrimination between the study groups. Additionally, greater dispersion is observed among the samples from the gastric cancer patient group compared to the healthy participants group, which is anticipated give the individual phenotypes for diseased patients (Figure 9).

**Figure 10:**
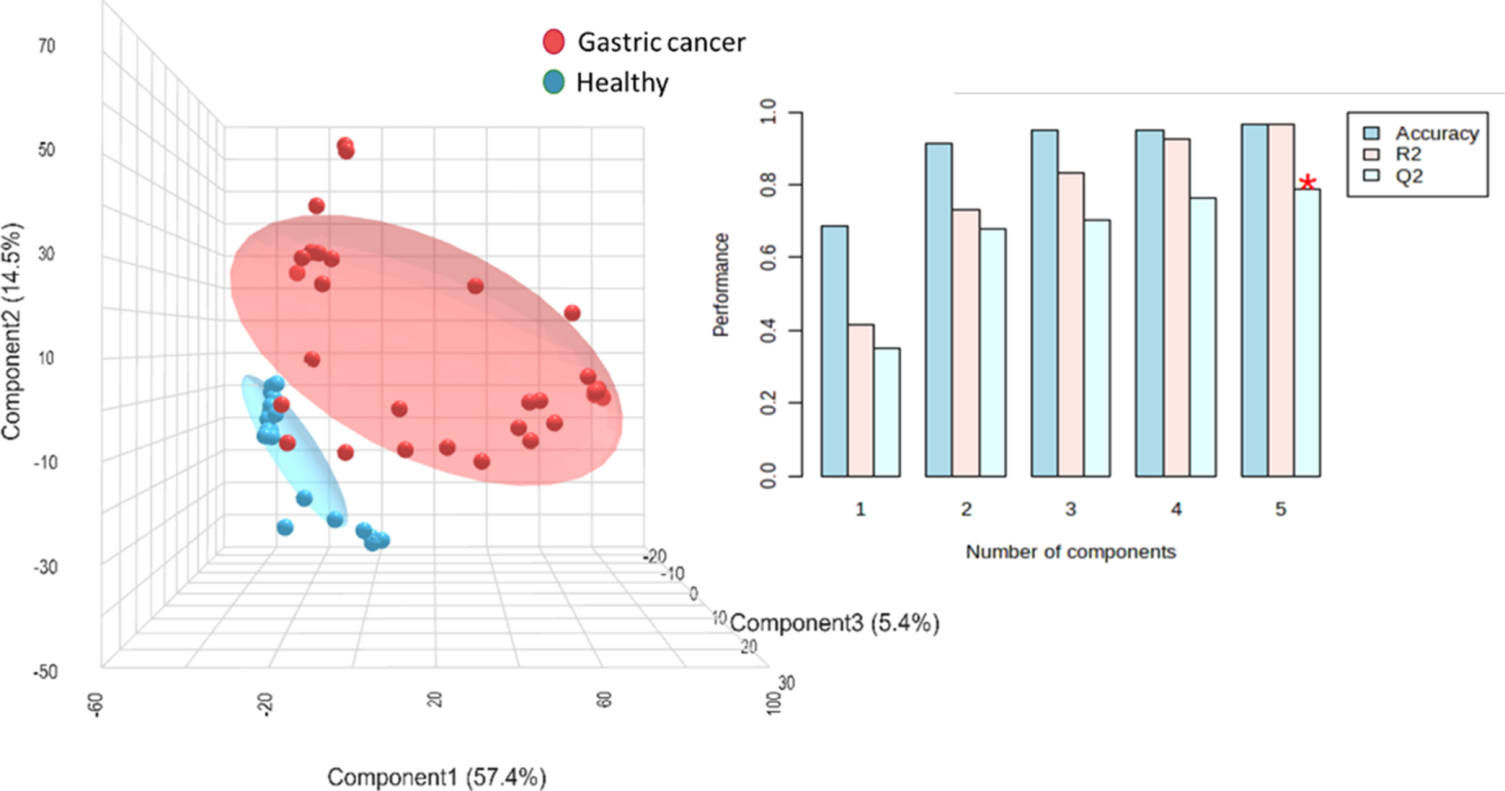
Partial least-squares-discriminant analysis (PLS-DA) and cross-validation for the evaluation of the IR-eNose system performance in exhaled breath from gastric cancer patients and healthy participants.

To further amplify the model separation, PLS-DA, followed by a 5-fold cross-validation and a permutation test to validate the outcome, was applied (Figure 10). The results for the cross-validation indicate that the model performance by PLS-DA is high; this explains the high variability between the groups and the high prediction parameters and thus, high model accuracy. Table S3 shows the PLS-DA cross-validation details; it is important to note that PLS-DA tends to overfit the model, by looking to sharpen the model separation; a value of *R*^2^ above 0.7 would be considered as a substantial predictive ability of the model; similarly, *Q*^2^ values should be close to the *R*^2^ value in order to indicate that the data are fitting the model; therefore, these results indicate that the model is not overfitted and the variability between groups is high. The cross-validation test from the PLS-DA model indicates an accuracy from 0.96 (where 1 is the maximum value) through five components.

**Figure 11:**
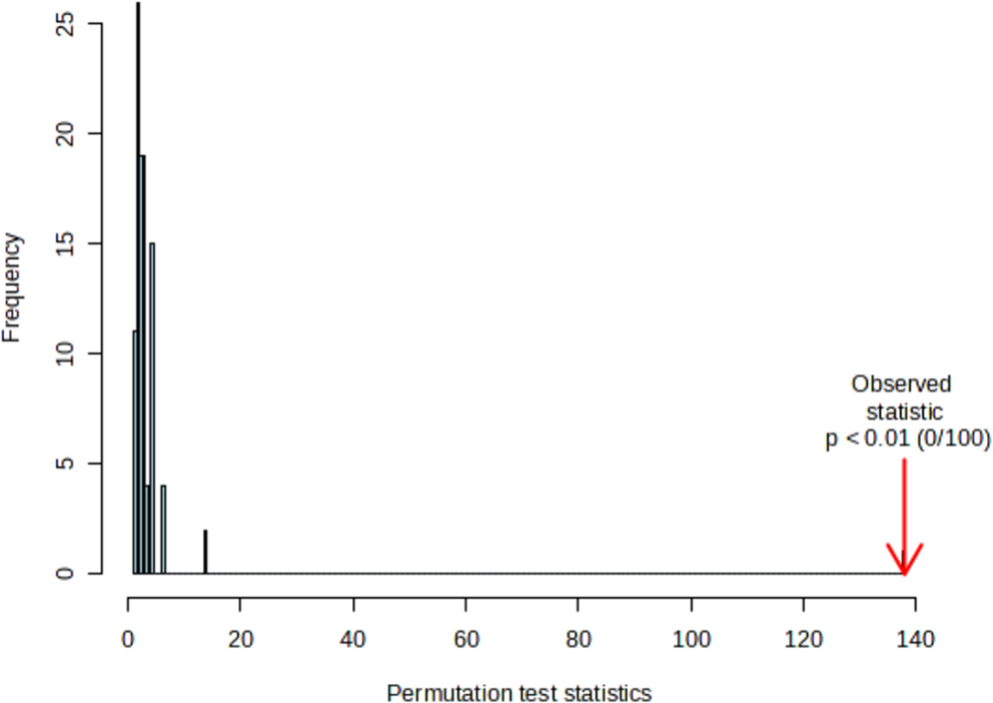
Permutation test for PLS-DA from the IR-eNose system feasibility study in exhaled breath of gastric cancer patients and healthy participants.

A permutation analysis was performed in order to further validate the PLS-DA (Figure 11). This permutation test is used to evaluate whether the class assignment is correct or incorrect. The histogram shows the distribution by the permuted samples, and the arrow represents the original sample; the further away from the normal distribution is the arrow, the more significant the separation is. For this permutation test, several (50×) randomized submodels were used to compare the performance of each submodel with the original model performance. The results are as follows: the permutation score for each one of the submodels was less than the one shown by the original model score.

The results indicate that the proposed model presents a high degree of discrimination between the study groups and could therefore serve as a predictive model during future clinical studies. By integration of orthogonal technologies, the results clearly demonstrate the enhanced predictive precision facilitating the analysis of exhaled breath from patients with gastric cancer and healthy participants.

## Discussion

The results presented in this study for the IR-eNose system underline the significant potential of combining both infrared spectroscopy and electronic nose technologies for advanced exhaled breath analysis, particularly for screening and monitoring of gastric cancer. This innovative approach addresses the limitations of existing diagnostic methods, offering a noninvasive, sensitive, and specific tool for early detection of gastric cancer.

To the best of our knowledge, this study is the first to demonstrate the feasibility of using combined technologies as anorthogonal approach for the analysis of exhaled breath, laying the foundation for developing more effective diagnostic devices tailored for exhaled breath analysis, focusing on enhancing accuracy while ensuring portability.

The complementary strengths of each technology enhance the diagnostic capability during exhaled breath analysis. IR spectroscopy offers a robust technique for identifying specific compounds based on their unique spectral fingerprints, allowing for accurate quantification of VOCs. This high specificity enables the distinction between chemically similar compounds, which is crucial for precise diagnostics. Conversely, the eNose with its sensor array provides rapid detection, even at minimal concentrations. The integration of these technologies ensures that while the eNose identifies the presence of specific compounds, IR spectroscopy can confirm and quantify these findings, thereby minimizing the risk of cross-reactivity and false positives.

A first clinical feasibility study demonstrates the fundamental utility of the developed technology. The application of the IR-eNose system in a pilot clinical setting involving gastric cancer patients presented promising results. The chemometric analysis indicated significant differences in the breath profiles of gastric cancer patients compared to healthy participants, showcasing the system’s ability to accurately differentiate between these groups. This capability positions the IR-eNose system as a potential screening tool for gastric cancer, particularly relevant given the challenges faced by existing screening methodologies. The IR-eNose system demonstrates that the integration of orthogonal sensing techniques enhances the reliability in exhaled breath analysis, while its portability facilitates on-site testing. This noninvasive approach not only improves patient comfort and compliance but also promises rapid results, making it a more accessible option for early screening, especially in underserved populations with limited healthcare access.

Despite the promising results, the study also acknowledges several challenges that need to be addressed in future research. The effects of humidity present a significant concern as moisture in exhaled breath interferes with the analysis of VOCs, particularly in spectral regions influenced by water vapor. To address this, ongoing efforts focus on improving moisture management techniques and analytical methods, further enhancing VOC analysis. Additionally, although the pilot nature of the feasibility study provides valuable insights, further investigations are needed to substantiate the efficacy of the technology. Also, we acknowledge that flow rate variability is a known factor affecting MOX sensors and will explore additional measures to further mitigate its impact, such as enhancing the pump and valve control algorithm to adapt to slight deviations dynamically and incorporating flow rate monitoring sensors for real-time correction and compensation. Future research includes longitudinal studies to monitor the device performance over time, assess its ability to track disease progression or treatment response, and expand the patient cohort to further augment the chemometric analysis.

## Conclusions

The IR-eNose system developed in this study presents a pioneering advancement in exhaled breath analysis for screening and monitoring of gastric cancer but also for other diseases characterized by changes of the exhaled VOC biomarker patterns. This study demonstrates the synergistic potential of combining IR spectroscopy with eNose technologies resulting in a sensitive, specific, portable, and noninvasive diagnostic tool. The IR-eNose system effectively differentiates between the VOC profiles of gastric cancer patients and healthy individuals, highlighting its capability to potentially serve as an innovative screening method beyond clinical settings, which could potentially aid the healthcare personnel in timely decision-making and assist in monitoring disease progression. Furthermore, its portability and rapid testing features render it particularly beneficial for underserved populations with limited healthcare access. While the results are promising, future research should address challenges such as humidity effects on VOC analysis and expand the patient cohorts for comprehensive validation. Overall, the IR-eNose system represents a significant step toward improving early detection methodologies in gastric cancer with the potential for broader applications in other diseases.

## Supporting Information Available

Additional methodological information, experimental details, statistical results including photograph of the devices. (PDF)

## Abbreviations

IR: infrared
FTIR: Fourier transform infrared spectroscopy
eNose: electronic nose
VOCs: volatile organic compounds
PCA: principal component analysis
PC: principal component
PLS-DA: partial least-squares discriminant analysis
EGC: esophagogastroduodenoscopy
EUR: euro
GC-MS: gas chromatography–mass spectrometry
SIFT-MS: selected-ion flow-tube mass spectrometry
PTR-MS: proton-transfer-reaction mass spectrometry
IMS: ion mobility spectrometry
IR-eNose: infrared-spectroscopic electronic nose
MFCs: mass flow controllers
iHWG: substrate-integrated hollow waveguide
MOX: metal oxide sensors
GNP: gold nanoparticle sensors
MCT: mercury cadmium telluride
ACE: acetone
ACH: acetaldehyde
N-PEN: *n*-pentane
NO: nitric oxide
