# Supplementary material for: Infrared Spectroscopic Electronic Noses: An Innovative Approach for Exhaled Breath Sensing
Source: ACS Sens. 2025 Jan 8;10(1):427–38. doi: 10.1021/acssensors.4c02725 (PMC11773567; doi:10.1021/acssensors.4c02725)
Supplement: Supplementary file 1 — se4c02725_si_001.pdf [file se4c02725_si_001.pdf]

Supporting information

Infrared Spectroscopic Electronic Noses: An Innovative Approach for Exhaled Breath Sensing

Johannes Glöckler[1], Jan Mitrovics[4], Sara Beeken[4], Marcis Leja[5,6,7,8], Tesfalem Welearegay[14], Lars Österlund[14], Hossam Haick[9], Gidi Shani[9], Corrado Di Natale[10,11], Raúl Murillo[12,13], Gabriela Flores-Rangel[1], Francisco Bricio-Arzubide[2], Raul Pinilla[12,13], Rómulo Vargas[12,13], Carlos Saboya[12,13], Boris Mizaikoff[1,3] and Lorena Díaz de León-Martínez[1,2*.]

[1]Institute of Analytical and Bioanalytical Chemistry, Ulm University, Albert-Einstein-Allee 11, 89081 Ulm, Germany.
[2]Breathlabs Inc. 77386, Spring, Texas, USA.
[3]Hahn-Schikard, Sedanstrasse 14, 89077 Ulm, Germany
[4]JLM Innovation GmbH, D-72070 Tübingen, Germany.
[5]Institute of Clinical and Preventive Medicine, University of Latvia, LV-1586 Riga, Latvia.
[6]Faculty of Medicine, University of Latvia, LV-1586 Riga, Latvia.
[7]Riga East University Hospital, LV-1038 Riga, Latvia.
[8]Digestive Diseases Centre GASTRO, LV-1079 Riga, Latvia.
[9]Laboratory for Nanomaterial-Based Devices, Technion—Israel Institute of Technology, Haifa 3200003, Israel.
[10]Department of Electronic Engineering, University of Rome Tor Vergata, 00133, Roma, Italy;
[11]Interdepartmental Center for Volatilomics, "A. D'Amico", University of Rome Tor Vergata, 00133, Rome, Italy.
[12] Centro Javeriano de Oncología, Hospital Universitario San Ignacio, 110231, Bogotá, Colombia.
[13]Facultad de Medicina, Pontificia Universidad Javeriana, 110231, Bogotá, Colombia.
[14] Department of Materials Science and Engineering. The Angstrom Laboratory Uppsala University, 752 37, Uppsala, Sweden.

Corresponding Author

* Lorena Díaz de León-Martínez. Institute of Analytical and Bioanalytical Chemistry, Ulm University, Albert-Einstein-Allee 11, 89081 Ulm, Germany & Breathlabs Inc. Spring, Texas, USA. E-mail address: lorena.diaz-de-leon@uni-ulm.de & lorena@breathlabs.com.

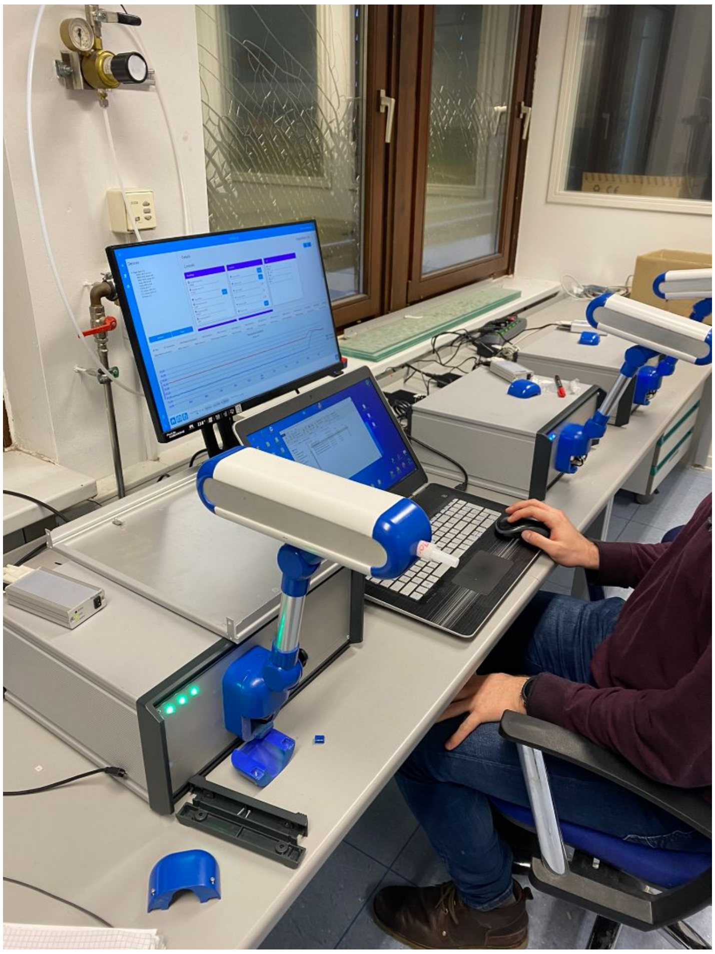

Figure 1 supplementary material. Fully functioning IR-eNose devices.

Table 1 supplementary material. Commercial MOX sensors in the IR-eNose devices.

**Analog Sensors (2X)**	CCS801	CCS801	MICS4514 Ox	MICS 4514 Red	MICS6814 NH3	MICS6814 Ox	MICS6814 Red	TGS8100
**Digital Sensors (2X)**	BME680	CCS811	IDTA	IDTB	SGP30 Ethanol	SGP30 H2		

Table 2 supplementary material. Acetone calibration parameters for each sensor.

SENSOR	INTERCEPT	SLOPE	CORRELATION COEFFICIENT	R^2
S1	2.51734E-05	6.00175E-07	0.933176646	0.8708187
S2	2.82776E-05	5.75054E-07	0.874958453	0.7655523
S3	1.94018E-06	-5.7748E-09	-0.623887012	0.389235
S4	6.09606E-06	3.96379E-08	0.632319441	0.3998279
S5	2.22007E-06	-6.48603E-09	-0.613290791	0.3761256
S6	5.28891E-06	2.62888E-08	0.544609948	0.2966

S7	1.20644E-06	5.90836E-09	0.997623122	0.9952519
S8	1.023E-06	5.94326E-09	0.998736633	0.9974749
S9	1.85825E-05	1.18099E-08	0.987898193	0.9759428
S10	1.61964E-05	5.95859E-10	0.815130337	0.6644375
S11	1.82313E-05	5.90901E-09	0.900243514	0.8104384
S12	1.63047E-05	-1.46008E-09	-0.867874094	0.7532054
S13	1.6939E-05	-4.60227E-09	-0.839628616	0.7049762
S14	1.6312E-05	-1.42597E-09	-0.849464452	0.7215899
S15	1.67313E-05	-3.32623E-09	-0.85747612	0.7352653
S16	1.62547E-05	-9.76727E-10	-0.855891017	0.7325494
S17	1.67298E-05	-3.34488E-09	-0.870441403	0.7576682
S18	4.57913E-07	4.94565E-09	0.977214148	0.9549475
S19	1.92467E-06	8.47827E-08	0.998106441	0.9962165
S20	1.16839E-05	2.14543E-08	0.166613394	0.02776
S21	4.9248E-06	1.43485E-07	0.964560799	0.9303775
S22	1.02929E-06	-8.40999E-09	-0.866525615	0.7508666
S23	6.51849E-06	7.12231E-08	0.988363824	0.976863
S24	1.29467E-06	8.22452E-08	0.998774821	0.9975511
S25	1.19865E-05	-1.6621E-08	-0.147682533	0.0218101

Table 3 supplementary material. Acetaldehyde calibration parameters for each sensor.

SENSOR	INTERCEPT	SLOPE	CORRELATION COEFFICIENT	R^2
S1	1.63097E-05	1.46815E-07	0.981231522	0.962815299
S2	1.94257E-05	2.71052E-07	0.994871449	0.9897692
S3	4.3285E-07	-3.17071E-09	-0.789835811	0.623840608
S4	4.57184E-06	2.42932E-08	0.950566111	0.903575932
S5	4.81873E-07	-3.63398E-09	-0.746110658	0.556681114
S6	3.65025E-06	1.58082E-08	0.956027042	0.913987706
S7	1.31396E-06	-8.89641E-09	-0.887686423	0.787987186
S8	1.1105E-06	-6.85129E-09	-0.862294441	0.743551702
S9	1.75875E-05	4.81975E-09	0.843877188	0.712128708
S10	1.61358E-05	2.60985E-10	0.827342105	0.684494958
S11	1.74607E-05	5.11496E-09	0.868542293	0.754365714
S12	1.6094E-05	-1.34005E-11	-0.848594283	0.720112257
S13	1.61237E-05	-9.71226E-10	-0.702216889	0.493108559
S14	1.60941E-05	-1.65535E-11	-0.894196751	0.799587829
S15	1.61111E-05	-8.24651E-11	-0.894820779	0.800704226
S16	1.61009E-05	-1.34119E-11	-0.848594283	0.720112257
S17	1.61107E-05	-8.11084E-11	-0.877116258	0.769332929
S18	4.56963E-07	3.63948E-09	0.985821683	0.971844391
S19	3.34149E-06	1.40233E-08	0.998921256	0.997843676
S20	5.27151E-06	-9.14415E-08	-0.988495788	0.977123923
S21	7.05826E-06	8.01399E-08	0.984685518	0.96960557
S22	4.9909E-08	-5.76155E-10	-0.92863507	0.862363094
S23	7.0013E-06	4.04107E-08	0.980935517	0.962234489
S24	1.48125E-06	7.49288E-08	0.934746453	0.873750932
S25	5.83094E-06	-9.35765E-08	-0.995724624	0.991467526

Table 4 supplementary material. N-pentane calibration parameters for each sensor.

SENSOR	INTERCEPT	SLOPE	CORRELATION COEFFICIENT	R^2
S1	1.91653E-05	1.33707E-08	0.986511926	0.97320578
S2	0.000609093	7.55273E-07	0.957310734	0.916443841
S3	1.04809E-06	2.36057E-09	0.92164845	0.849435866
S4	5.37439E-06	7.66343E-09	0.968546469	0.938082262
S5	1.21946E-06	2.93365E-09	0.905332023	0.819626071
S6	3.83846E-06	1.59647E-08	0.774635032	0.600059433
S7	1.19325E-06	2.91774E-09	0.936413157	0.8768696
S8	1.03183E-06	2.07347E-09	0.976298919	0.953159579
S9	1.79429E-05	7.76851E-09	0.888798056	0.789961984
S10	1.60612E-05	8.16866E-12	0.947192113	0.897172899
S11	1.6121E-05	4.17099E-10	0.896892038	0.804415327
S12	1.61367E-05	2.61186E-10	0.90359096	0.816476622
S13	1.6306E-05	1.07989E-09	0.906888432	0.822446628
S14	1.61378E-05	2.80413E-10	0.905866095	0.820593382
S15	1.62924E-05	6.85479E-10	0.898470741	0.807249672
S16	1.61394E-05	1.73264E-10	0.885094104	0.783391573
S17	1.62905E-05	6.93152E-10	0.898142283	0.806659561
S18	4.26814E-07	4.08548E-10	0.931542921	0.867772214
S19	1.67886E-06	8.08975E-09	0.95540804	0.912804523
S20	4.65683E-06	4.56728E-08	0.925207024	0.856008038
S21	3.7765E-06	4.07403E-08	0.976205469	0.952977117
S22	3.29741E-07	9.79438E-10	0.733588808	0.53815254
S23	6.29547E-06	2.09312E-08	0.879195296	0.772984369
S24	1.23895E-06	5.69167E-09	0.898724202	0.807705191
S25	5.84114E-06	2.89648E-08	0.783399292	0.61371445

Table 5 supplementary material. Nitric Oxide calibration parameters for each sensor

SENSOR	INTERCEPT	SLOPE	CORRELATION COEFFICIENT	R^2
S1	8.1872E-06	1.84553E-08	0.994123122	0.988280782
S2	9.64955E-05	7.80134E-07	0.936727242	0.877457927
S3	9.57804E-06	1.52301E-08	0.994852657	0.989731808
S4	0.000101679	7.89003E-07	0.939904425	0.883420329
S5	3.1951E-07	1.9516E-09	0.914679685	0.836638926
S6	3.68098E-06	1.29888E-08	0.912364189	0.832408414
S7	3.22234E-07	1.85885E-09	0.922458198	0.850929126
S8	3.07011E-06	1.0434E-08	0.905225671	0.819433515
S9	9.77144E-07	8.40832E-10	0.700844849	0.491183502
S10	8.80102E-07	9.39354E-11	0.195543468	0.038237248
S11	1.76757E-05	4.53032E-09	0.873457836	0.762928592
S12	1.61392E-05	2.03526E-10	0.857621494	0.735514628
S13	1.60823E-05	3.36688E-12	0.846153846	0.715976331

S14	1.60611E-05	1.6027E-12	0.466325473	0.217459447
S15	1.61089E-05	1.77463E-10	0.859250409	0.738311265
S16	1.74274E-05	4.01726E-09	0.876999307	0.769127784
S17	1.60966E-05	5.24993E-11	0.889636954	0.791453909
S18	1.61287E-05	4.20395E-10	0.8736158	0.763204567
S19	1.60968E-05	5.57958E-11	0.873714318	0.76337671
S20	1.61399E-05	4.6874E-10	0.8805272	0.775328151
S21	1.61045E-05	6.43626E-11	0.881847525	0.777655057
S22	1.61389E-05	4.58472E-10	0.879640998	0.773768286
S23	4.08136E-07	-1.2575E-09	-0.986458377	0.973100131
S24	1.68862E-06	4.42274E-09	0.939637631	0.882918878
S25	1.18501E-06	1.16206E-08	0.95218364	0.906653684
S26	4.98189E-06	-4.02585E-09	-0.99812692	0.996257349
S27	5.00265E-08	6.10123E-10	0.890663393	0.79328128
S28	4.87421E-06	1.42642E-08	0.924693673	0.855058389
S29	1.13526E-06	3.33776E-09	0.947858743	0.898436196
S30	1.20729E-06	9.42358E-09	0.993363794	0.986771627

Table 6 supplementary material. PLS-DA cross-validation details from the eNose calibration model.

Measure	1 comps	2 comps	3 comps	4 comps	5 comps
**Accuracy**	0.60897	0.82821	0.90513	0.90513	0.92051
**R2**	0.36742	0.73151	0.74868	0.76172	0.84668
**Q2**	0.33882	0.68473	0.67536	0.74943	0.70092

Table 7 supplementary material. Random Forest classification results from the IR-eNose device chemometric model

	ACE	ACH	NO	N-PEN	Classification error
**ACE**	14	0	0	0	0
**ACH**	0	14	0	0	0
**NO**	0	0	24	1	0.04
**N-PEN**	0	1	1	9	0.182

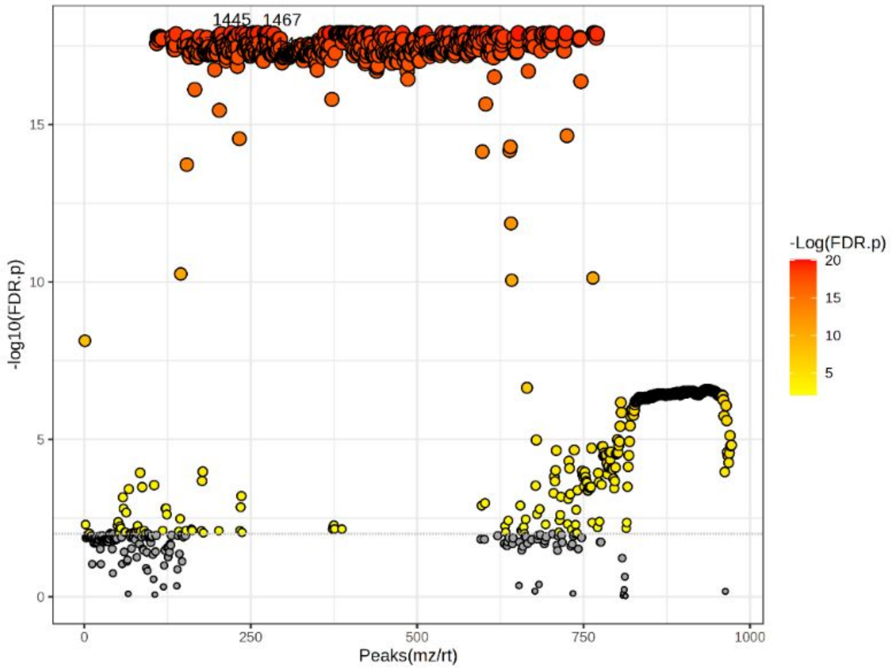

Figure 2 supplementary material. Volcano plot from T-test for statistically significant features in the IR spectra from the groups of study.

Table 8 supplementary material. PLS-DA cross-validation details from the IR-eNose device chemometric model.

Measure	1 comps	2 comps	3 comps	4 comps	5 comps
**Accuracy**	0.686336	0.91515	0.95	0.95	0.96667
**R2**	0.41589	0.73216	0.83144	0.92458	0.96777
**Q2**	0.34982	0.67385	0.70309	0.7628	0.78804
